# Identification of cuproptosis-related molecular subtypes as a biomarker for differentiating active from latent tuberculosis in children

**DOI:** 10.1186/s12864-023-09491-2

**Published:** 2023-07-01

**Authors:** Liang Chen, Jie Hua, Xiaopu He

**Affiliations:** 1 Department of Infectious Diseases, Nanjing Lishui People’s Hospital, Zhongda Hospital Lishui Branch, Southeast University, No.86, Chongwen Street, Lishui District, Nanjing City, 211002 China; 2 Department of Gastroenterology, The First Affiliated Hospital of Nanjing Medical University, Nanjing, China; 3 Department of Geriatric Gastroenterology, The First Affiliated Hospital of Nanjing Medical University, Nanjing, China

**Keywords:** Cuproptosis, Molecular subtype, Active tuberculosis, Latent tuberculosis infection, Prediction model

## Abstract

**Background:**

Cell death plays a crucial role in the progression of active tuberculosis (ATB) from latent infection (LTBI). Cuproptosis, a novel programmed cell death, has been reported to be associated with the pathology of various diseases. We aimed to identify cuproptosis-related molecular subtypes as biomarkers for distinguishing ATB from LTBI in pediatric patients.

**Method:**

The expression profiles of cuproptosis regulators and immune characteristics in pediatric patients with ATB and LTBI were analyzed based on GSE39939 downloaded from the Gene Expression Omnibus. From the 52 ATB samples, we investigated the molecular subtypes based on differentially expressed cuproptosis-related genes (DE-CRGs) via consensus clustering and related immune cell infiltration. Subtype-specific differentially expressed genes (DEGs) were found using the weighted gene co-expression network analysis. The optimum machine model was then determined by comparing the performance of the eXtreme Gradient Boost (XGB), the random forest model (RF), the general linear model (GLM), and the support vector machine model (SVM). Nomogram and test datasets (GSE39940) were used to verify the prediction accuracy.

**Results:**

Nine DE-CRGs (*NFE2L2, NLRP3, FDX1, LIPT1, PDHB, MTF1, GLS, DBT,* and *DLST*) associated with active immune responses were ascertained between ATB and LTBI patients. Two cuproptosis-related molecular subtypes were defined in ATB pediatrics. Single sample gene set enrichment analysis suggested that compared with Subtype 2, Subtype 1 was characterized by decreased lymphocytes and increased inflammatory activation. Gene set variation analysis showed that cluster-specific DEGs in Subtype 1 were closely associated with immune and inflammation responses and energy and amino acids metabolism. The SVM model exhibited the best discriminative performance with a higher area under the curve (AUC = 0.983) and relatively lower root mean square and residual error. A final 5-gene-based (*MAN1C1, DKFZP434N035, SIRT4, BPGM,* and *APBA2*) SVM model was created, demonstrating satisfactory performance in the test datasets (AUC = 0.905). The decision curve analysis and nomogram calibration curve also revealed the accuracy of differentiating ATB from LTBI in children.

**Conclusion:**

Our study suggested that cuproptosis might be associated with the immunopathology of *Mycobacterium tuberculosis* infection in children. Additionally, we built a satisfactory prediction model to assess the cuproptosis subtype risk in ATB, which can be used as a reliable biomarker for the distinguishment between pediatric ATB and LTBI.

**Supplementary Information:**

The online version contains supplementary material available at 10.1186/s12864-023-09491-2.

## Background

Tuberculosis, an infection caused by *Mycobacterium tuberculosis* (Mtb), is the most lethal pathogen-related cause of death and one of the leading global causes of human mortality [1]. Tuberculosis affects 500,000 to 1,000,000 children annually, with ~ 226,000 fatalities worldwide [2]. 5–15% of the estimated 2–3 billion Mtb-infected individuals are expected to develop tuberculosis at some point in their lives, with the highest risk among young children [3]. The treatment of tuberculosis can be complex and time-consuming, resulting in low patient compliance, especially among pediatric populations. The factors that ultimately govern the transition between active tuberculosis (ATB) and latent tuberculosis infection (LTBI) remain fully clarified. The clinical differentiation between these two disease states remains challenging despite being critical to providing patients with appropriate treatments to curtail further tuberculosis spread. The two most common methods for determining tuberculosis infection status are the tuberculin skin test and the interferon γ release assay. Still, neither method can distinguish between ATB and LTBI in all cases [4]. The results may be non-reactive in malnourished children with tuberculosis or a comorbid human immunodeficiency virus infection [5]. Consequently, there is an urgent need to develop alternative biomarkers that can reliably differentiate between these two types of tuberculosis infection.

Immune responses are the predominant factor in the control of Mtb infection [6]. Previous research has shown that the death of host cells is involved in regulating Mtb infections [7]. Increasing evidence indicates that the crosstalk between cell death and host immune responses is essential for developing ATB following LTBI [8, 9]. Recently, cuproptosis, a novel form of programmed cell death distinct from the well-known regulated cell death processes, was discovered [10]. This cell death is copper-dependent, may be controlled, and is strongly associated with mitochondrial respiration. The direct binding of copper causes Cuprotosis to the lipoacylated component of the tricarboxylic acid cycle, which results in the aggregation of lipoacylated proteins and the loss of Fe-S cluster proteins, ultimately causing protein toxic stress and cuproptosis. According to research, the interaction between copper and Mtb is crucial for regulating Mtb infection [11]. In addition, many studies indicate that mitochondrial dysfunction-induced deficiencies in energy metabolism and oxidative stress contribute to the activation of LTBI. Consequently, it is acceptable to conclude that cuproptosis is closely related to ATB's emergence from LTBI.

**Fig. 1:**
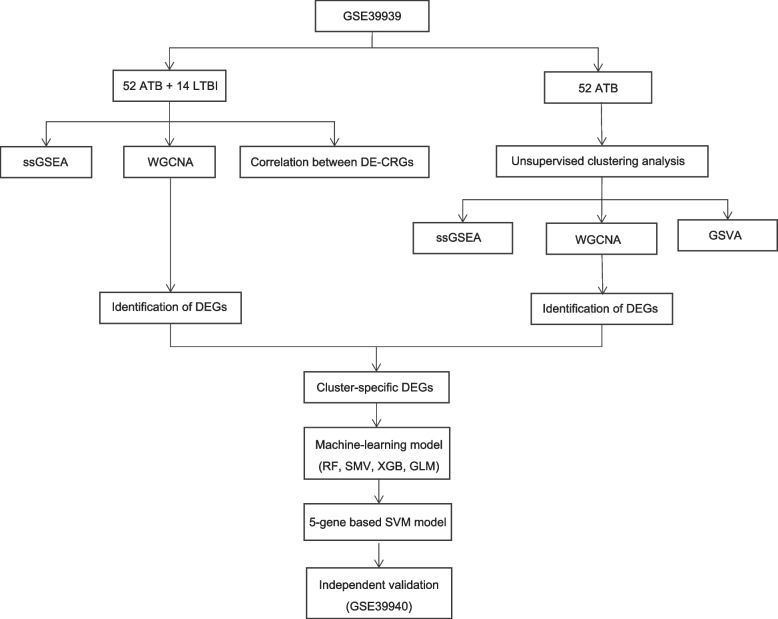
Shows the flow chart for the present study

However, cuproptosis regulation mechanisms in Mtb infection are not well understood. The variation in Mtb infection may be explained by further elucidating the molecular properties of cuproptosis-related genes (CRGs). This study aimed to extensively investigate the differentially expressed CRGs (DE-CRGs) and immunological features in ATB children. The DE-CRG expression patterns were then used to divide ATB patients into two groups, and the genes characteristic of each subtype were used to determine which biological activities and pathways were enriched. As a bonus, by contrasting several machine learning methods [eXtreme Gradient Boost (XGB), the Random Forest (RF) model, the Generalized Linear Model (GLM), and the Support Vector Machine (SVM) algorithms], a prediction model was built to reveal patients with distinct molecular subtypes. The accuracy of the prediction model was checked using an external dataset, a calibration curve (CC), a nomogram, and a Decision Curve Analysis (DCA). Figure 1 represents the flowchart for this analysis.

## Results

### Comparison of CRGs expression levels and immune cells infiltration between ATB and LTBI children

**Fig. 2:**
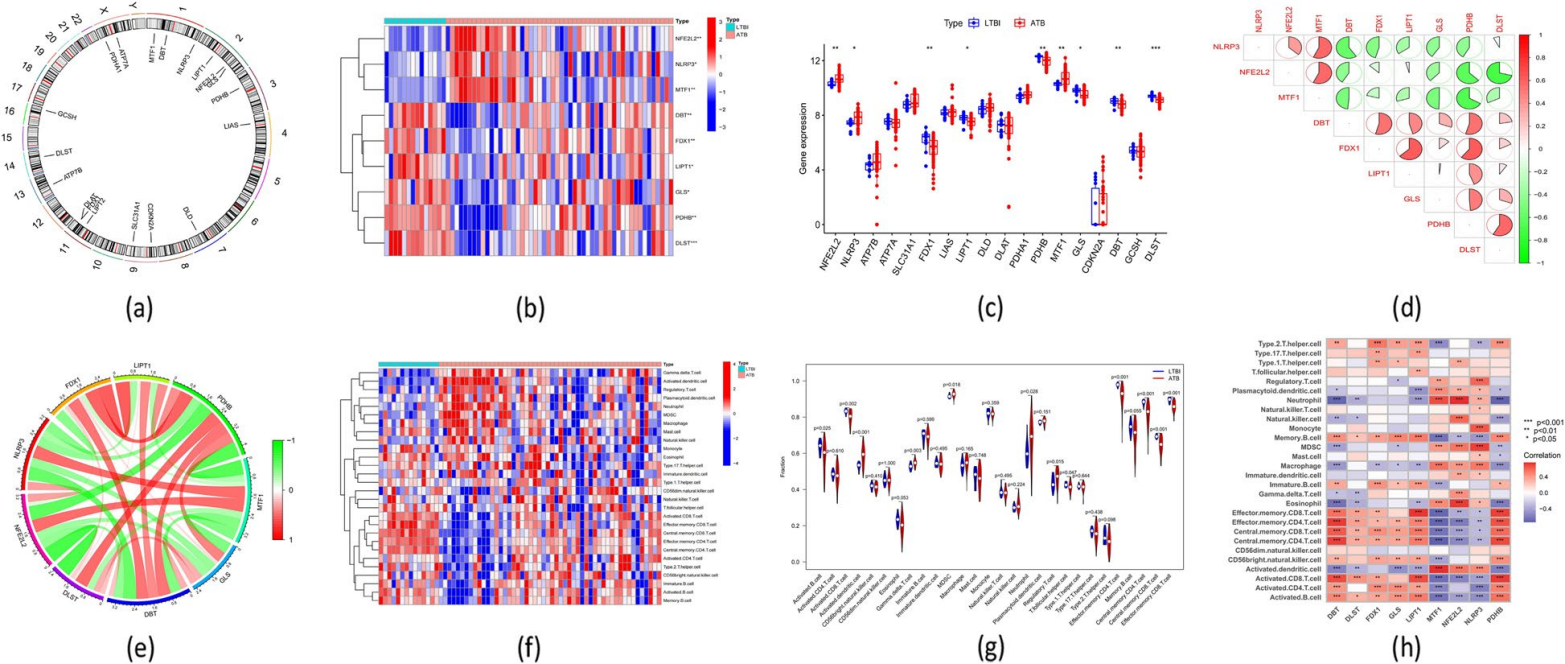
Shows the identification of CRGs that have been dysregulated in pediatric patients with ATB. **a** The specific positions on the chromosomes of the 19 CRGs. **b** The heatmap comprised representations of the expression patterns for 19 CRGs. Each CRG is represented along the X-axis, while the relative expression levels of genes are displayed along the Y-axis. **c** Boxplots illustrated the differences in the expression of 19 CRGs between patients with ATB and LTBI. **d** An examination of the correlations between the 9 CRGs and differential expression. The color red denotes a positive connection, whereas the color green represents a negative correlation. The areas of the pie chart corresponded to the calculated correlation coefficients. **e** A gene association network diagram shows the 9 CRGs that have differential expression. **f** The differences in the relative abundances of 28 infiltrating immune cells between patients with ATB and LTBI. **g** Boxplots illustrated 28 immune cell infiltration disparities between individuals diagnosed with ATB and LTBI. Each immune cell is shown along the X-axis, and the relative risk score is displayed along the Y-axis. **h** The correlation between the infiltrating immune cells and the 9 CRGs with differential expression was studied. *p < 0.05, **p < 0.01, ***p < 0.001

The location of 19 CRGs on chromosomes can be seen in Fig. 2a. A total of 9 CRGs (*NFE2L2, NLRP3, FDX1, LIPT1, PDHB, MTF1, GLS, DBT,* and *DLST*) were determined as DE-CRGs between pediatric ATB and LTBI patients by Wilcoxon test. The expression levels of *MTF1, NFE2L2*, and *NLRP3* were higher, while the *FDX1, LIPT1, PDHB, GLS, DBT,* and *DLST* were lower in the ATB group than LTBI group (Fig. 2b-c). The correlation of the seven differentially expressed CRGs (DE-CRGs) is presented in Fig. 2d. Some had positive relationships (e.g., *NLRP3* and *NFE2L2* with *MTF1, DBT* with *FDX1, LIPT1,* and *PDHB*), while some had negative associations (e.g., *MTF1* with *DBT*, *GLS,* and *PDHB*). The association between these differentially expressed CRGs was also shown using a gene connection network diagram (Fig. 2e).

The Single-sample Gene Set Enrichment Analysis (ssGSEA) algorithm was employed to assess the relative immune cell infiltration in the training dataset. The results revealed the suppression of lymphocytes, which was mainly presented by a lower infiltration of memory CD8 T cells, activated CD8 T cells, memory CD4 T cells and activated CD4 T, and B cells and activation of inflammatory and myeloid cells (e.g., a considerably elevated infiltration of neutrophils, dendritic cells, and monocytes) in ATB patients (Fig. 2f-g). Meanwhile, correlation analysis showed that the nine DE-CRGs correlated with the immune cells, suggesting that CRGs may be the key factors in controlling ATB's molecular and immune infiltration status progressed from LTBI (Fig. 2h).

### Identification of cuproptosis subtypes in ATB pediatrics

**Fig. 3:**
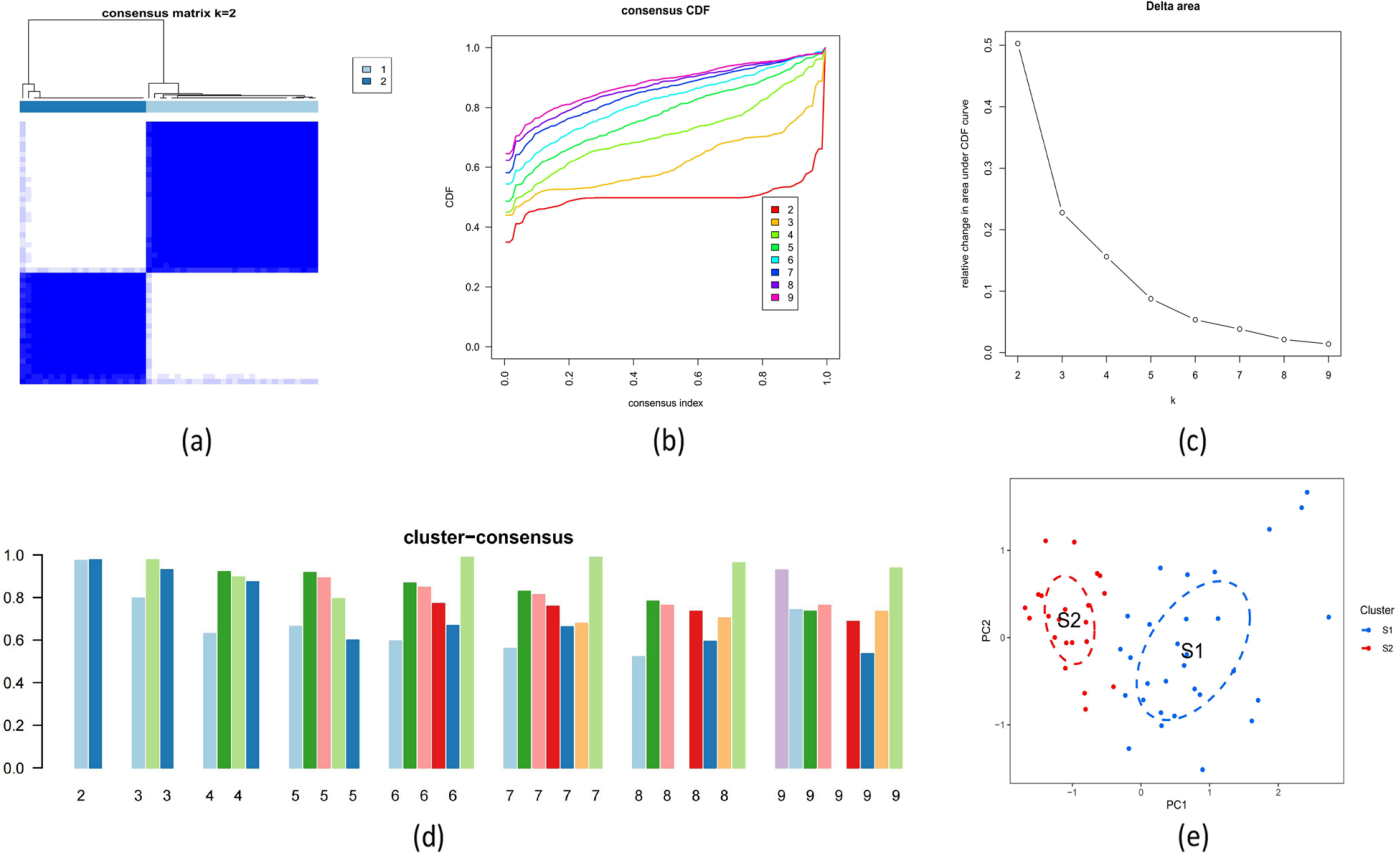
Displays the identification of ATB cuproptosis-related molecular subtypes. **a** Clustering consensus matrix for k = 2. **b** CDF delta area curves.**c** The consensus clustering score. **d** The non-negative matrix heatmap. **e** The distribution of two subtypes is visualized using t-SNE

A consensus clustering approach was used to classify the 52 samples from ATB patients centered on the expression profiles of 9 DE-CRGs, to elucidate the cuproptosis-related expression patterns in ATB. At k = 2, the cluster numbers were most stable, while the Cumulative Distribution Function (CDF) curves ranged between 0.2 and 0.8 on average (Fig. 3a-b). The area under the CDF curves showed the separation between the two CDFs (k and k-1) for k = 2 to 9 (Fig. 3c). Moreover, the reliability score of each subtype was > 0.8 only at k = 2 (Fig. 3d). In a nutshell, the t-Distributed Stochastic Neighbor Embedding (tSNE) analysis showed that the two subtypes were distinct (Fig. 3e).

### Differentiation of cuproptosis regulators, immune infiltration characteristics, and functional annotation between cuproptosis subtypes

**Fig. 4:**
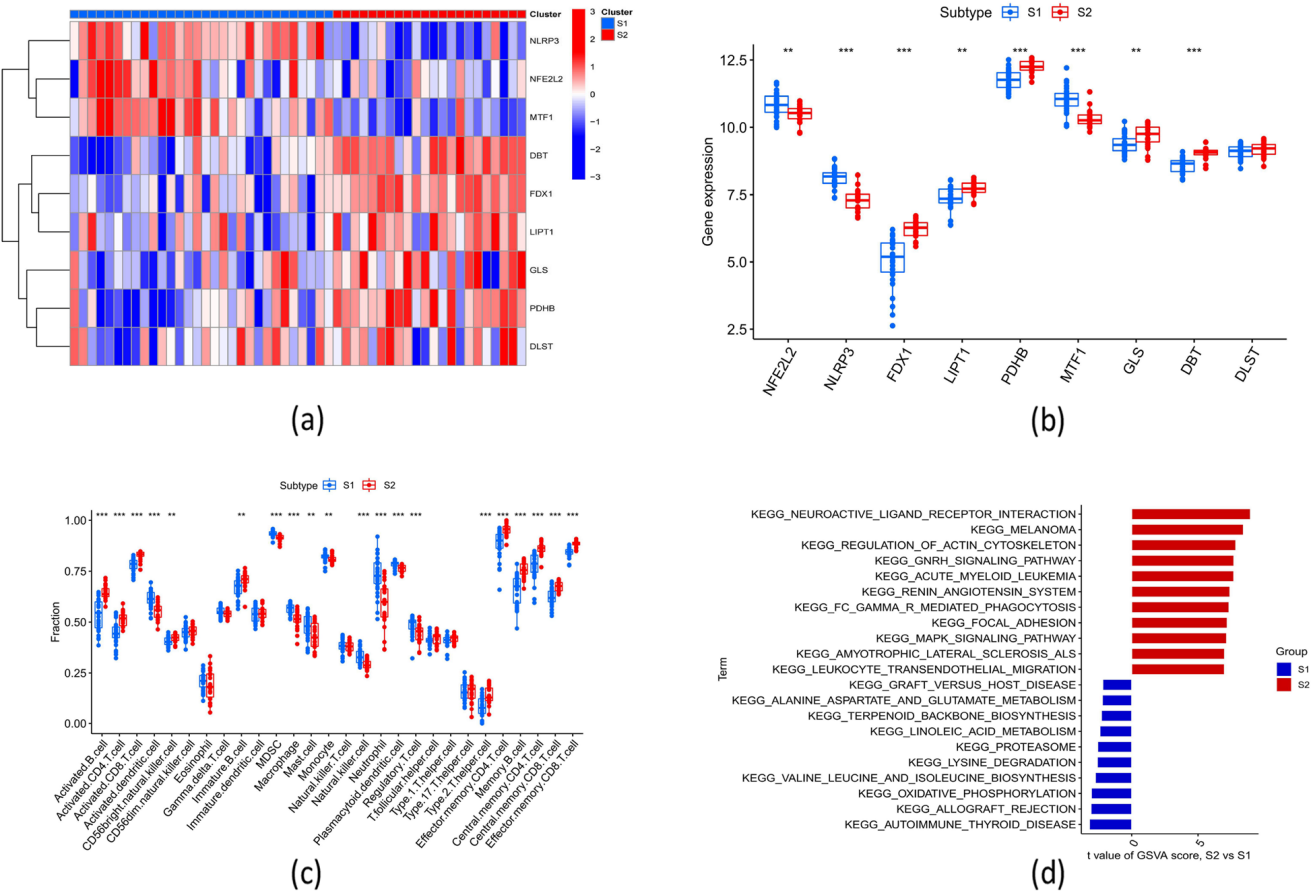
Shows the molecular and immunological differences between the two cuproptosis subtypes. **a** Heatmap of 9 DE-CRG expression patterns between two cuproptosis subtypes. **b** Boxplots comparing the expression of 9 DE-CRGs in the two cuproptosis subtypes. The X-axis indicates each DE-CRG, and the Y-axis depicts gene expression levels. **c** Boxplots demonstrating the variations in the infiltration of 28 immune cells between the two cuproptosis subtypes. The X-axis depicts each immune cell, while the Y-axis displays the relative risk score. **d** GSVA t-value ranking differences in hallmark pathway activities between Subtype 1 and Subtype 2 samples. *p < 0.05, **p < 0.01, ***p < 0.001

The expression levels of *MTF1, NFE2L2*, and *NLRP3* were higher in CRGs subtype 1, while the expression levels of *LIPT1, PDHB, GLS,* and *DBT* were higher in subtype 2 (Fig. 4a-b). The immune infiltration analysis results showed that the infiltration of lymphocytes (e.g., activated and memory CD8 T cells, activated and memory CD4 T cells, and B cells) was lower. In contrast, that of inflammatory and myeloid cells (e.g., monocytes, neutrophils, macrophages, and dendritic cells) was higher in subtype 1 (Fig. 4c).

The Gene Set Variation Analysis (GSVA) indicated that the pathways of immune and inflammation responses (KEGG_AUTOIMMUNE_THYROID_DISEASE, KEGG_ALLOGRAFT_REJECTION), metabolism of energy (KEGG_OXIDATIVE_PHOSPHORYLATION) and amino acid (KEGG_LYSINE_DEGRADATION, KEGG_VALINE_LEUCINE_AND_ISOLEUCINE_BIOSYNTHESIS) were upregulated in subtype 1. While the pathways of neuro activation (KEGG_NEUROACTIVE_LIGAND_RECEPTOR_INTERACTION), cell cycle, ERK and PI3K signaling (KEGG_REGULATION_OF_ACTIN_CYTOSKELETON, KEGG_MELANOMA) were upregulated in subtype 2 (Fig. 4d).

### Gene modules screening and co-expression network construction

**Fig. 5:**
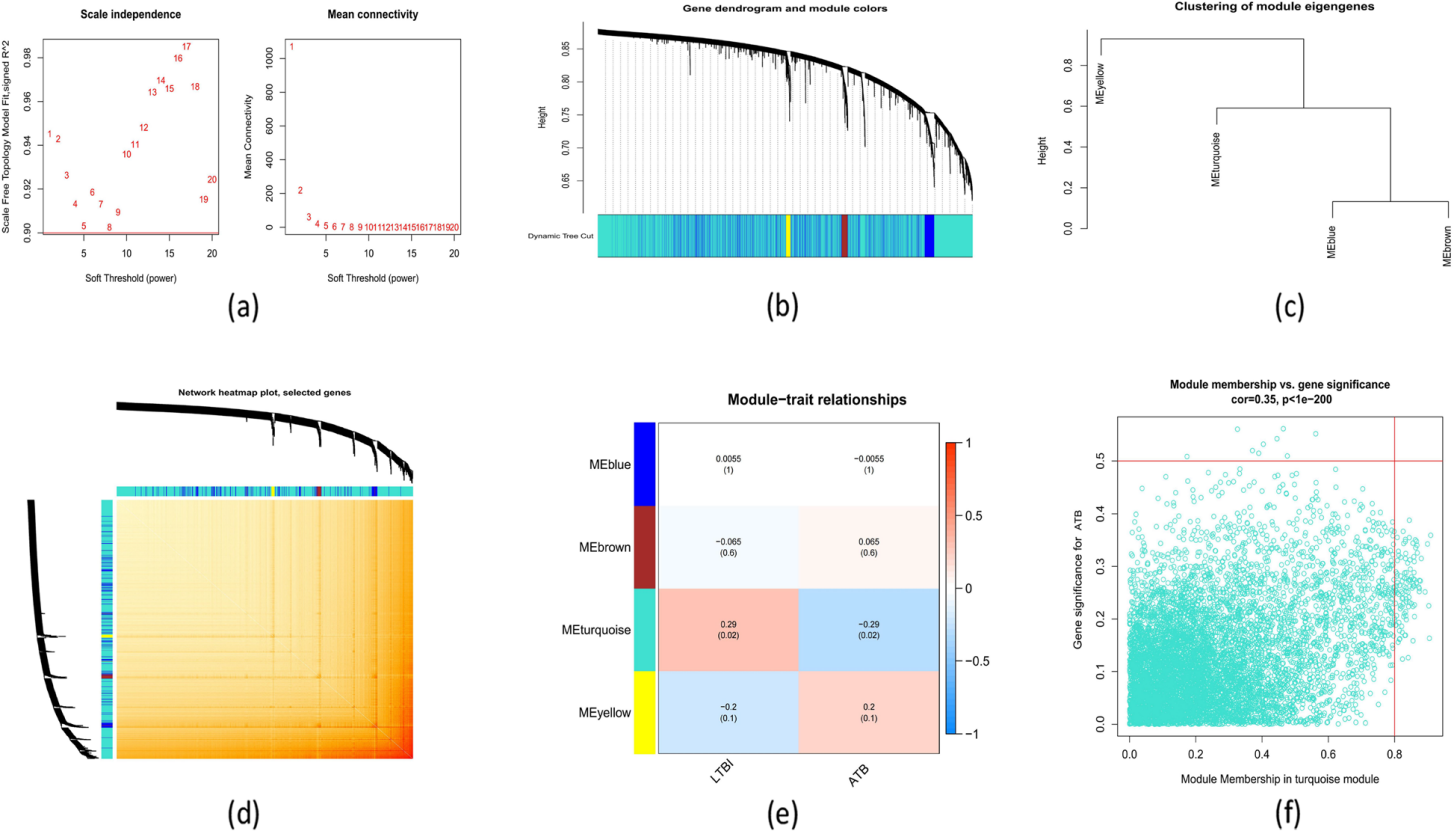
Shows a co-expression network of differentially expressed genes in aTB patients. **a** Establishing an appropriate threshold power. **b**Co-expression module cluster tree dendrogram. Each color reflects different modules of co-expression. **c** Illustration of module eigengene clustering. **d** A heatmap that shows the correlations between the 4 modules. **e** A correlation analysis between the clinical status and the module eigengenes. Each column denotes a clinical status, whereas each row indicates a module. **f** A scatter plot showing the relationship between the gene importance for aTB and turquoise module membership

The training set (GSE39939) was used to construct a co-expression network by Weighted Gene Co-Expression Network Analysis (WGCNA) to assess the critical modules associated with ATB. We analyzed the importance of hub gene expression level diagnosis and assessment using a cut height of 0.25 and a soft-thresholding power of 1. We eventually settled on the three components shown in Fig. 5a-d. Using heat map profiling, we discovered a correlation between the modules and clinical sample attributes, and we were able to quantify this correlation by looking at the relationship between module eigengene (ME) values and sample traits. The turquoise module was shown to be the most closely related to the disease stage (cor = 0.29, *p* = 0.02) (Fig. 5e). Moreover, a significant correlation (cor = 0.35, *p* < 1E-200) was found between the turquoise module and module-related genes (Fig. 5f).

**Fig. 6:**
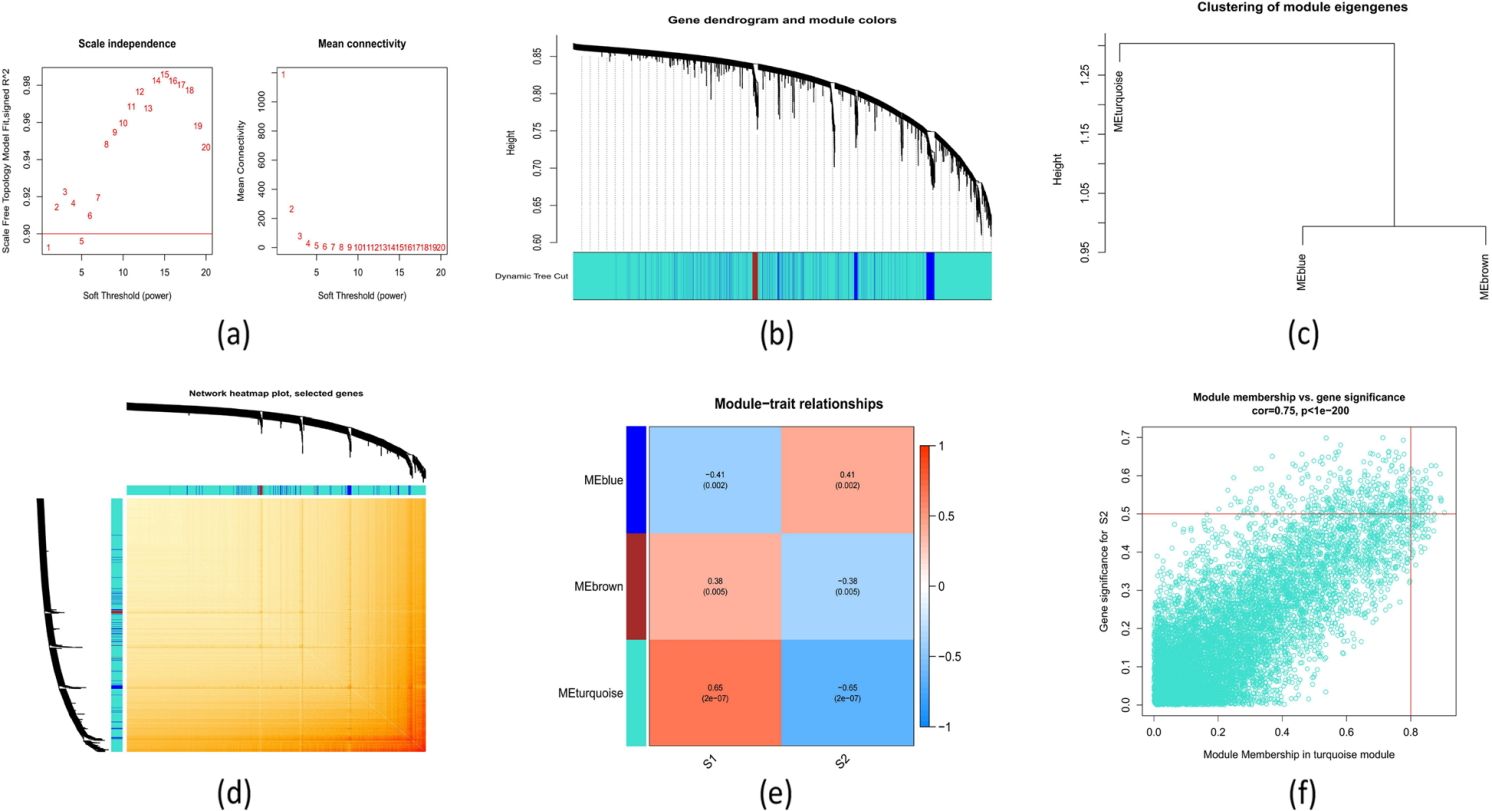
Demonstrates a co-expression network of genes differentially expressed in the two cuproptosis subtypes. **a** Applying a minimal threshold power. **b** Co-expression module cluster tree dendrogram. Multiple colors indicate different modules of co-expression. **c** Representation of module eigengene clustering. **d** A heatmap depicting the correlations between 3 modules. **e** Representation of the correlation analysis of module eigengenes and clinical status. Each row represents a module and each column a clinical status. **f** Scatter plot of turquoise module membership versus gene importance for Subtype 1

Using the WGCNA technique, we also evaluated the crucial gene modules related to cuproptosis subtypes. According to our screening, the best soft threshold values for building a scale-free network were β = 1 and R^2^ = 0.9 (Fig. 6a). In particular, three modules were recognized as important, and the TOM of all genes associated with those modules are shown in a heatmap (Fig. 6b–d). Analysis of module-clinical characteristics (Subtype 1 and Subtype 2) showed a strong positive link between the turquoise module and module-related genes (cor = 0.75, *p* < 1E-200) (Fig. 6e) and a significant correlation between the turquoise module and ATB subtypes (cor = 0.65, *p* = 2E-7) (Fig. 6f).

### The development and evaluation of machine learning models

**Fig. 7:**
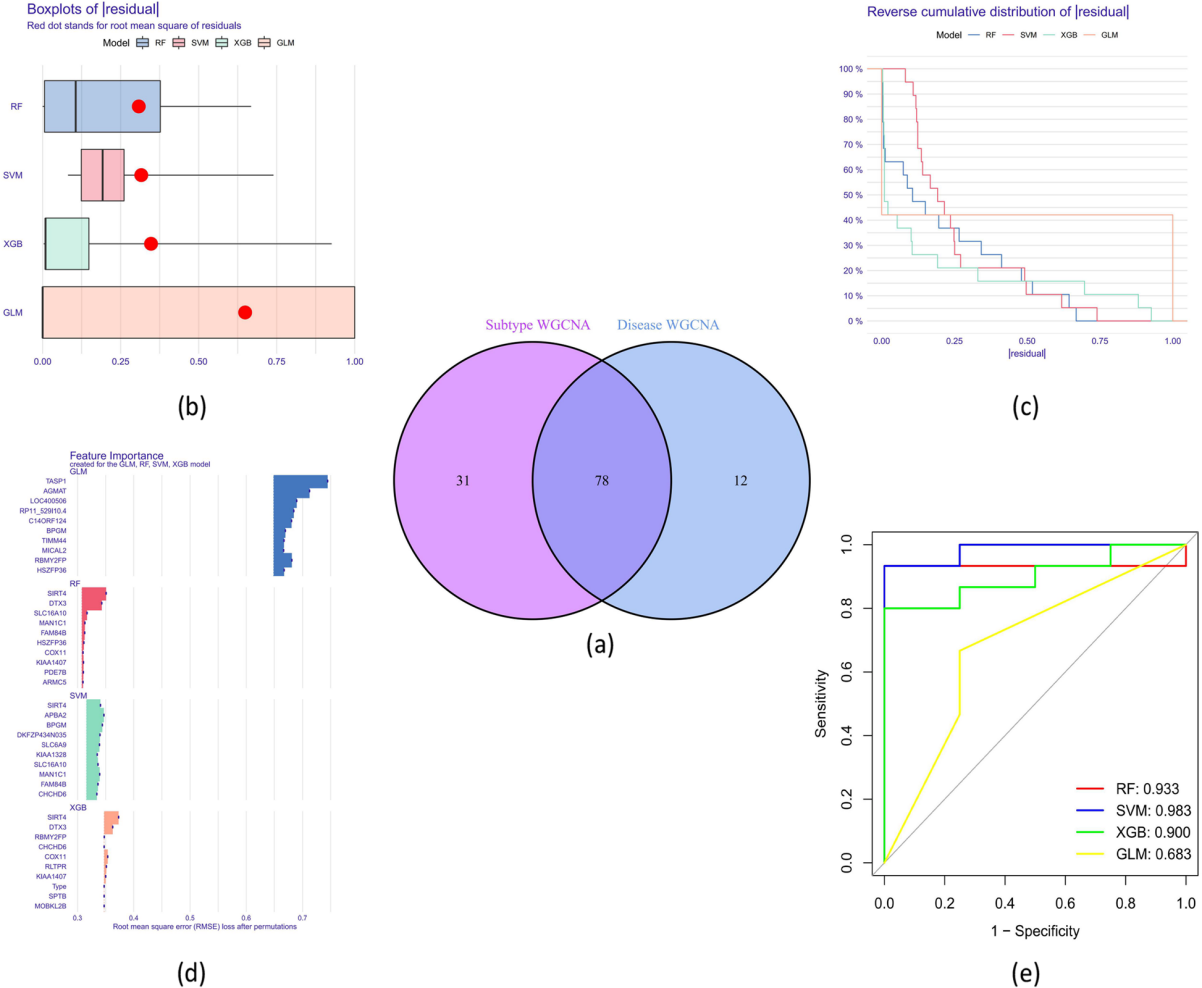
Shows the development of machine models and the identification of subtype-specific DEGs. **a** The connections between module-related genes in the training dataset and cuproptosis subtype-related genes. **b** The distribution of cumulative residuals for each machine learning model. **c** Boxplots displayed each machine learning model's residuals. The root mean square of residuals (RMSE) was shown as a red dot. **d** The crucial components of the machine models for RF, SVM, GLM, and XGB. **e** ROC analysis of the training dataset's four machine learning models

Comparing the module-related genes of cuproptosis subtypes to those of ATB and LTBI patients, we could identify a total of 78 cluster-specific DEGs (Supplementary file 3) (Fig. 7a). Based on the expression patterns of 78 subtype-specific DEGs in the ATB patients from the training dataset, we constructed four proven machine-learning models (RF, GLM, SVM, and XGB) (Fig. 7b-d). Outperforming the RF (AUC = 0.933), XGB (AUC = 0.900), and GLM (AUC = 0.683), the SVM machine learning model had the largest AUC of 0.983 (Fig. 7e).

**Fig. 8:**
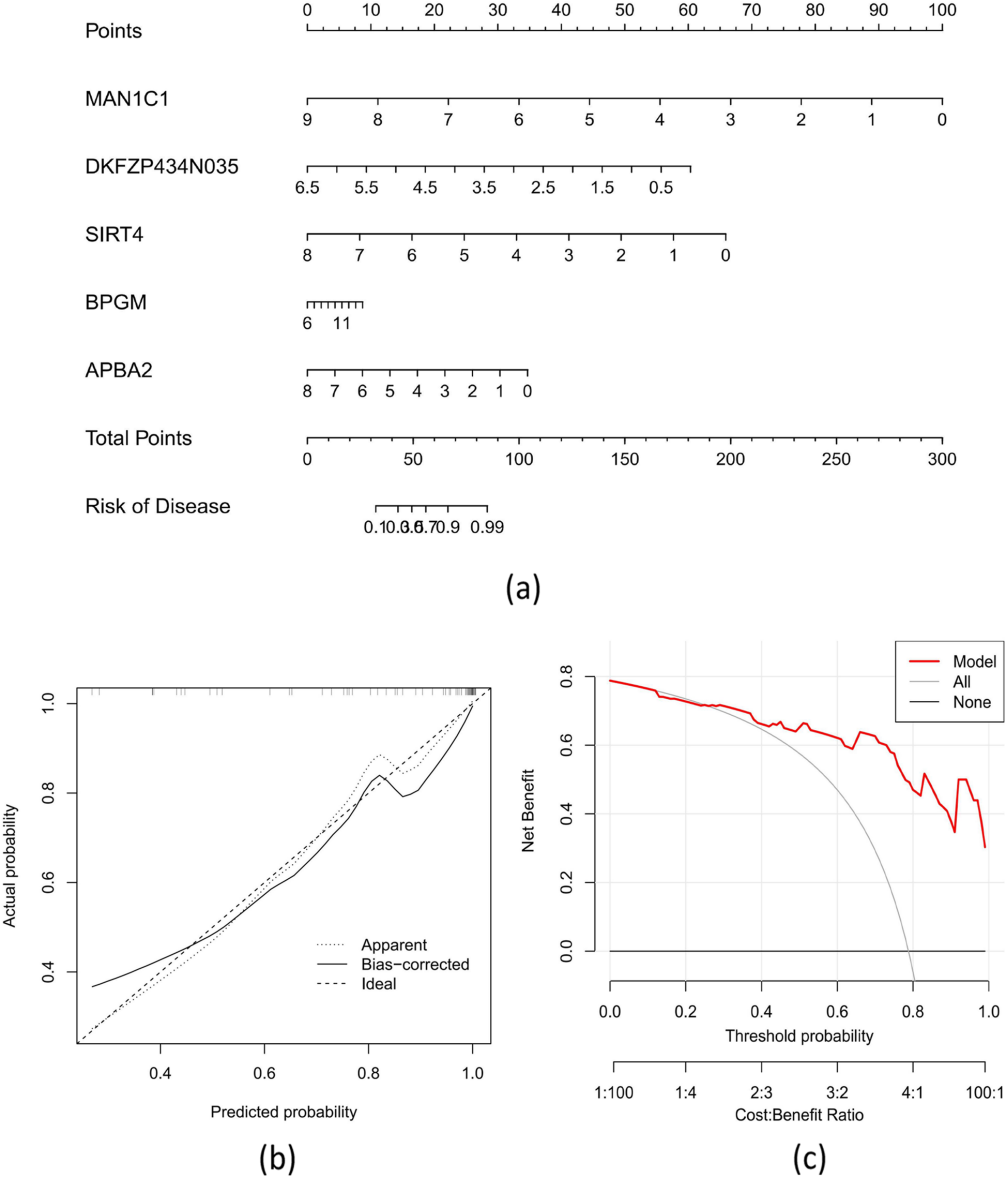
Shows the development of a nomogram model **a** Construction of a nomogram for estimating the risk of AD clusters using the SVM model based on 5 genes and establishing a calibration curve (**b**) and a DCA (**c**) to evaluate the predictive efficiency of the nomogram model

**Fig. 9:**
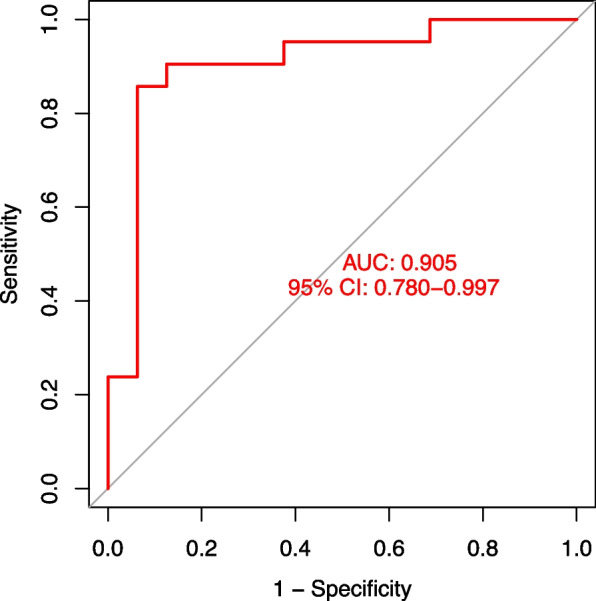
Shows the ROC analysis of the 5-gene SVM model using the test dataset and fivefold cross-validation

We initially developed a nomogram to quantify the probability of cuproptosis subtypes in 52 ATB patients to evaluate the SVM model's prediction efficacy (Fig. 8a). The DCA shows that our nomogram has a high degree of accuracy, and the CC shows a very small difference between the observed and projected risks of ATB subtypes (Fig. 8b-c). Then, we used the test data (GSE39940) to validate our 5-gene prediction model (*MAN1C1, DKFZP434N035, SIRT4, BPGM*, and *APBA2*). The 5-gene prediction model performed well in ROC analyses, with an AUC of 0.905 (Fig. 9).

## Discussion

Host cell death controls Mtb outgrowth and restricts infection dissemination [7–9]. Apoptosis during Mtb infection is a defense mechanism for cells confronted with intracellular pathogens [7]. In contrast, necrotic cell death is considered host-detrimental since it facilitates mycobacterial spread [12]. Cuproptosis, a recently found programmed cell death, differs in the initiating stimuli, intermediate activation events, and end effectors from other forms of cell death and is involved in the progression of tumor and neurodegenerative diseases [13, 14]. By far, no studies have been reported on cuproptosis-related genes' role in Mtb infection. Recent transcriptomic research efforts have highlighted a range of genes and gene expression patterns related to the stage of Mtb infection [15, 16]. We systemically explored the cuproptosis-related gene expression profiles in Mtb infection individuals for the first time. In line with previous reports [17, 18], our study showed that the immune status of ATB patients was characterized by the suppression of lymphocytes, which presented by a significant drop in throughout CD4 T cells, CD8 + T cells, and B cells, together with the activation of myeloid and inflammation cells (e.g., macrophages. neutrophils, and monocytes). T cells and their antibodies may impact the course of an Mtb infection by affecting granuloma development [19]. Limiting lymphocyte responses is therefore associated with uncontrolled Mtb infection and the transition from LTBI to ATB [20]. Extensive inflammatory responses, including DCs, monocytes, macrophages, and neutrophils, are generated in ATB patients in response to the disseminated bacteria and accompanying tissue damage. Further, we discovered that the expression of some DE-CRGs (e.g., *NFE2L2, NLRP3*, and *MTF1*) was upregulated in ATB pediatric patients, which were negatively associated with lymphocytes, while positively correlated to myeloid and inflammation cells; some DE-CRGs (e.g., *DBT, DLSTFD, LIPT1*, and *PDHB*) were downregulated, which were positively associated with lymphocytes, and negatively related to inflammation cells. The dysregulation of CRGs and their association with immune cell populations highlight the potentially important role that copper-induced cell death may participate in the pathogenesis of LTBI progressing to ATB in children.

We used unsupervised cluster analysis to highlight the various cuproptosis regulation patterns in pediatric ATB patients based on the expression landscapes of nine DE-CRGs tested and discovered two unique cuproptosis-related subtypes. ICI analysis showed that Subtype 1 was characterized by the suppression of lymphocytes and activation of myeloid and inflammatory cells. Subtype-specific DEGs indicated that Subtype 1 was primarily enriched in regulating immune and inflammation responses and energy and amino acid metabolism. These results implied that Subtype 1 was more closely related to the immunopathology of ATB.

Over the last two decades, effective and precise computer methods have been developed to mine the growing mountain of biological data for insights [21]. It has been shown that machine learning models may provide an appropriate platform for merging data from disparate sources to comprehend the intertwining nature of genetic, environmental, and demographic information in the development of different diseases [22]. Thus, compared to univariate analysis, the outcomes of these multifactorial studies are more accurate and credible. Using the expression patterns of subtype-specific DE-CRGs, we developed a prediction model for pediatric ATB using four machine-learning classifiers (RF, GLM, SVM, and XGB). RF uses several decision trees for classification or regression prediction [23]. Using the SVM technique, one may create a hyperplane with a maximum margin to discriminate between negative and positive examples [24]. In order to examine the association between normally distributed dependent characteristics and categorical or continuous independent data, GLM was developed as an extension of multiple linear regression models [25]. XGB is a gradient-boosting-based ensemble of boosted trees that allows for a thorough analysis of the trade-offs between model complexity and classification error [26]. Ultimately, it was shown that SVM-based machine learning had the greatest AUC in predicting the subtypes of ATB patients.

Afterward, a 5-gene-based RF model was constructed by selecting five important genes (*MAN1C1, DKFZP434N035, SIRT4, BPGM*, and *APBA2*). A previous study showed that α-mannosidase I (*MAN1C1*) participates in cellular immunity during some chronic infections, such as HBV. The upregulation of a-mannosidase I expression manipulates the pathogens to escape immune recognition by DC-SIGN, inhibiting an efficient immune response and clearance of the etiologies [27]. SIRT4 resides within the mitochondria and belongs to the sirtuin (SIRT) family, with ADP-ribosyltransferase, lipoamidase, substrate-specific deacetylase, and deacetylase functions [28]. *SIRT4* has also been linked to the defense of cells against bacteria. *SIRT4* overexpression in LPS-treated cells boosted steroidogenesis and lowered apoptosis, promoting Mtb infection propagation and LTBI activation [29]. Moreover, SIRT4 was also found to suppress the anti-inflammatory activity, contributing to the inflammation response in ATB [30]. BPGM is a glycolytic enzyme that converts 1,3-diphosphoglycerate to 2,3-diphosphoglycerate (2,3-DPG), a tiny molecule that modulates the oxygen affinity of hemoglobin and is highly expressed in red blood cells [31]. It was reported that the *BPGM* gene expression was significantly upregulated in some form of inflammation and infection [32]. Additionally, *BPGM* is related to T- and B-cell receptor signaling pathways and neutrophilia [33]. However, *DKFZP434N035* is an uncharacterized gene. Our results suggested that the *DKFZP434N035* might play a vital role in Mtb infection by affecting immunity or cell death. Additionally, using the five genes, we created a nomogram model for identifying ATB subtypes. Our prediction model had excellent predictive efficacy, proving its relevance for clinical applications.

This research has clear limitations. First, although attempts were made to locate all related publicly accessible datasets, the sample size for these analyses was relatively small, which may have hampered the precision of these conclusions. The link between CRGs and immune cells revealed in this research should be classified as a statistical correlation rather than a causative one. Also, it is unclear whether these host variables are exclusive to Mtb infection. Finally, microarrays have several disadvantages (e.g., not a whole genome analysis, high background signal levels, not quantitative, and an inability to detect alternative splicing). In order to determine the processes behind the pathophysiology of pediatric ATB advanced from LTBI, further in vitro and in vivo investigations examining the activity of these CRGs are required.

## Conclusions

In conclusion, we comprehensively evaluated the cuproptosis-related gene expression levels and immune cell infiltration in pediatrics with ATB and LTBI. Moreover, we constructed a 5-gene-based model to predict the cuproptosis subtypes of ATB risk in children. These results suggest that CRGs can be used as potential biomarkers for differentiating ATB from LTBI in pediatric patients. Future studies are recommended to concentrate on the mechanisms of cuproptosis in conjunction with the immunopathology of Mtb infection.

## Methods

### Data source

For the present study, data were downloaded from the NCBI-GEO database (http://www.ncbi.nlm.nih.gov/geo). Inclusion criteria: 1) Patients < 15 years of age; 2) Sample collection prior to the initiation of anti-mycobacterial treatment; 3) Negative for human immunodeficiency virus (HIV). Two datasets (GSE39939 and GSE39940) were selected for analysis based on these three criteria. The GSE39939 microarray dataset concluded whole-blood samples from 52 and 14 pediatric ATB and LTBI patients, which was used as the training dataset for constructing a prediction model. The GSE39940 microarray dataset of whole-blood samples from 52 and 54 pediatric ATB and LTBI patients validated the prediction model's performance.

### Evaluation of CRGs expression and immune cell infiltration (ICI) between ATB and LTBI pediatric patients

To further understand the biological roles of cuproptosis regulators in ATB, we used the training dataset to compare the expression patterns of 19 CRGs between ATB and LTBI in pediatric patients (Supplementary file 1). In order to determine the relative infiltration levels of immune cells in the training set, the ssGSEA algorithm was applied (Supplementary file 2). Violin plots depict the varying degrees of expression produced by invading immune cells. Spearman correlations between immune-invading cells and CRGs were shown using the R 'ggplot2' package (version 4.2.2).

### Unsupervised clustering of ATB patients

Based on the 9 DE-CRGs expression profiles, the "ConsensusClusterPlus" R package (version 4.2.1) was employed to perform the unsupervised clustering analysis and classify the 52 samples from ATB cases into different subtypes using the k-means algorithm with 1,000 iterations. After carefully considering the consensus matrix, the CDF curve, and the consistent cluster score (> 0.8), we settled on a maximum subtype number of k (k = 9).

### GSVA

To further understand how the various subtypes of CRGs vary in terms of enriched gene sets, the “GSVA” R package (version 4.2.2) was employed. In order to continue the GSVA analysis, the “c2.cp.kegg.symbols” file [34] was downloaded from the MSigDB internet database. Differentially expressed biological functions and pathways were determined by comparing GSVA scores across CRG clusters using the "limma" R package (version 4.2.2). Significant changes were defined as |t value of GSVA score|> 2 and p-value < 0.05.

### WGCNA

The R package “WGCNA” (version 4.2.2) was used to conduct WGCNA and find co-expression modules. The top 25% of most variable genes were included in subsequent WGCNA analyses to provide reliable quality findings. We first determined the optimum soft power to create a weighted adjacency matrix, which was then converted into a topological overlap matrix (TOM). When the minimum module size was set to 100, modules were produced using the TOM dissimilarity measure (1-TOM) in conjunction with the hierarchical clustering tree algorithm. Each module was assigned a random color. The eigengenes of each module reflected the overarching patterns of gene expression in that particular module. Modular significance (MS) demonstrated the link between modules and disease conditions. When discussing the relationship between a gene and its clinical manifestation, the term “gene significance” (GS) was coined.

### Construction of predictive model based on multiple machine learning methods

We used the "caret" R packages (version 4.2.3) to create machine learning models such as RF, GLM, SVM, and XGB based on two classes of CRGs (the codes of the models can be found in Supplementary file 4). The distinct subtypes were used as the response variable, while subtype-specific differentially expressed genes were selected as the explanatory factors. 70% of the 52 ATB samples were part of the training set, while the remaining 30% was a validation set. All models were run with their default settings and evaluated using fivefold cross-validation, and their parameters were automatically tweaked using a grid search using the caret package. The "DALEX" package (version 4.2.2) was executed to understand the models mentioned above and show their residual distribution and feature significance. The AUC was plotted using the "pROC" R program (version 4.2.1). As a result, the most suited model was established, and the top five factors were considered ATB's most significant predictor genes. In the end, the diagnostic model's accuracy was tested using ROC curve analysis.

### Construction and validation of a nomogram model

Using the “rms” R package (version 4.2.3), a nomogram was developed to evaluate the frequency of ATB subtypes. Each predictor has an associated score, and the "total score" is the total of all the aforementioned predictors' scores. Using a combination of DCA and the CC, we were able to calculate an approximation of the nomogram model's predictive ability. The ROC analyses were used in the test dataset (GSE39940) to validate the prediction model's ability to discriminate between ATB and LTBI in pediatric patients. The "pROC" R package allowed for the visualization of ROC curves.

## Supplementary Information

**Additional file 1.**

**Additional file 2: Supplemenatry file 2.** Detailed results of ssGSEA algorithm in the training set.

**Additional file 3: Supplementary file3.** 78 cluster-specific DEGs.

**Additional file 4. **

## Data Availability

Publicly available datasets were analyzed in this study. These data can be found in GSE39939 (https://www.ncbi.nlm.nih.gov/geo/query/acc.cgi?acc=GSE39939), GSE 39940 (https://www.ncbi.nlm.nih.gov/geo/query/acc.cgi?acc=GSE39940).

## Abbreviations

Mtb: *Mycobacterium tuberculosis*
ATB: Active tuberculosis
LTBI: Latent tuberculosis infection
CRGs: Cuproptosis-related genes
GEO: Gene Expression Omnibus
DEGs: Differentially Expressed Genes
ssGSEA: Single-sample Gene Set Enrichment Analysis
GSVA: Gene Set Variation Analysis
WGCNA: Weighted Gene Co-Expression Network Analysis
ROC: Receiver Operating Characteristic Curve
AUC: Area Under the Curve
RF: Random Forest model
SVM: Support Vector Machine model
GLM: Generalized Linear Model
XGB: EXtreme Gradient Boosting
DCA: Decision Curve Analysis
CC: Calibration Curve

## References

[1] Gong W, Liang Y, Wu X (2018). The current status, challenges, and future developments of new tuberculosis vaccines. Hum Vaccin Immunother.

[2] Singhal T (2022). The new WHO consolidated guidelines for management of tuberculosis in children and adolescents: an appraisal. Indian J Pediatr.

[3] Carvalho I, Goletti D, Manga S, Silva DR, Manissero D, Migliori G (2018). Managing latent tuberculosis infection and tuberculosis in children. Pulmonology.

[4] Pai M, Behr M. Latent Mycobacterium tuberculosis Infection and Interferon-Gamma Release Assays. Microbiol Spectr. 2016;4(5):1–10. 10.1128/microbiolspec.TBTB2-0023-2016 27763261

[5] Jaganath D, Beaudry J, Salazar-Austin N (2022). Tuberculosis in Children. Infect Dis Clin North Am.

[6] Mayer-Barber KD, Barber DL (2015). Innate and Adaptive Cellular Immune Responses to Mycobacterium tuberculosis Infection. Cold Spring Harb Perspect Med.

[7] Lam A, Prabhu R, Gross CM, Riesenberg LA, Singh V, Aggarwal S (2017). Role of apoptosis and autophagy in tuberculosis. Am J Physiol Lung Cell Mol Physiol.

[8] Amaral EP, Costa DL, Namasivayam S, Riteau N, Kamenyeva O, Mittereder L, Mayer-Barber KD, Andrade BB, Sher A (2019). A major role for ferroptosis in Mycobacterium tuberculosis-induced cell death and tissue necrosis. J Exp Med.

[9] Scordo JM, Knoell DL, Torrelles JB (2016). Alveolar epithelial cells in mycobacterium tuberculosis Infection: active players or Innocent bystanders?. J Innate Immun.

[10] Tomioka H (2014). New approaches to tuberculosis–novel drugs based on drug targets related to toll-like receptors in macrophages. Curr Pharm Des.

[11] Tang D, Chen X, Kroemer G (2022). Cuproptosis: a copper-triggered modality of mitochondrial cell death. Cell Res.

[12] Darwin KH (2015). Mycobacterium tuberculosis and Copper: A Newly Appreciated Defense against an Old Foe?. J Biol Chem.

[13] Robinson N, Ganesan R, Hegedus C, Kovacs K, Kufer TA, Virag L (2019). Programmed necrotic cell death of macrophages: Focus on pyroptosis, necroptosis, and parthanatos. Redox Biol.

[14] Bian Z, Fan R, Xie L (2022). A novel cuproptosis-related prognostic gene signature and validation of differential expression in clear cell renal cell carcinoma. Genes (Basel).

[15] Lai Y, Lin C, Lin X, Wu L, Zhao Y, Lin F (2022). Identification and immunological characterization of cuproptosis-related molecular clusters in Alzheimer's disease. Front Aging Neurosci.

[16] Mulenga H, Musvosvi M, Mendelsohn SC, Penn-Nicholson A, KimbungMbandi S, Fiore-Gartland A, Tameris M, Mabwe S, Africa H, Bilek N (2021). Longitudinal dynamics of a blood transcriptomic signature of tuberculosis. Am J Respir Crit Care Med.

[17] Hoang LT, Jain P, Pillay TD, Tolosa-Wright M, Niazi U, Takwoingi Y, Halliday A, Berrocal-Almanza LC, Deeks JJ, Beverley P (2021). Transcriptomic signatures for diagnosing tuberculosis in clinical practice: a prospective, multicentre cohort study. Lancet Infect Dis.

[18] Berry MP, Graham CM, McNab FW, Xu Z, Bloch SA, Oni T, Wilkinson KA, Banchereau R, Skinner J, Wilkinson RJ (2010). An interferon-inducible neutrophil-driven blood transcriptional signature in human tuberculosis. Nature.

[19] Achkar JM, Chan J, Casadevall A (2015). B cells and antibodies in the defense against Mycobacterium tuberculosis infection. Immunol Rev.

[20] LindestamArlehamn CS, Lewinsohn D, Sette A, Lewinsohn D (2014). Antigens for CD4 and CD8 T cells in tuberculosis. Cold Spring Harb Perspect Med.

[21] Paulson LC (2018). Computational logic: its origins and applications. Proc Math Phys Eng Sci.

[22] Reel PS, Reel S, Pearson E, Trucco E, Jefferson E (2021). Using machine learning approaches for multi-omics data analysis: A review. Biotechnol Adv.

[23] Sarica A, Cerasa A, Quattrone A (2017). Random forest algorithm for the classification of neuroimaging data in alzheimer's disease: A systematic review. Front Aging Neurosci.

[24] Rjoob K, Bond R, Finlay D, McGilligan V, Leslie SJ, Rababah A, Iftikhar A, Guldenring D, Knoery C, McShane A (2022). Machine learning and the electrocardiogram over two decades: time series and meta-analysis of the algorithms, evaluation metrics and applications. Artif Intell Med.

[25] Schuemann J, Bassler N, Inaniwa T (2018). Computational models and tools. Med Phys.

[26] Saqib K, Khan AF, Butt ZA (2021). Machine learning methods for predicting postpartum depression: scoping review. JMIR Ment Health.

[27] Hu S, Jiang LB, Zou XJ, Yi W, Tian DY (2016). Hepatitis B virus upregulates host expression of alpha-1,2-mannosidases via the PPARalpha pathway. World J Gastroenterol.

[28] Min Z, Gao J, Yu Y (2018). The Roles of Mitochondrial SIRT4 in Cellular Metabolism. Front Endocrinol (Lausanne).

[29] Ramatchandirin B, Sadasivam M, Kannan A, Prahalathan C (2016). Sirtuin 4 Regulates Lipopolysaccharide Mediated Leydig Cell Dysfunction. J Cell Biochem.

[30] Das UN (2021). “Cell membrane theory of senescence” and the role of bioactive lipids in aging, and aging associated diseases and their therapeutic implications. Biomolecules.

[31] Lazana I, Mohamedali A, Smith F, de Lavallade H, McLornan D, Raj K (2021). Uniparental disomy (UPD) of a novel bisphosphoglycerate mutase (BPGM) mutation leading to erythrocytosis. Br J Haematol.

[32] Scicluna BP, van Vught LA, Zwinderman AH, Wiewel MA, Davenport EE, Burnham KL, Nurnberg P, Schultz MJ, Horn J, Cremer OL (2017). Classification of patients with sepsis according to blood genomic endotype: a prospective cohort study. Lancet Respir Med.

[33] Yin GQ, Zeng HX, Li ZL, Chen C, Zhong JY, Xiao MS, Zeng Q, Jiang WH, Wu PQ, Zeng JM (2021). Differential proteomic analysis of children infected with respiratory syncytial virus. Braz J Med Biol Res.

[34] Kanehisa M, Furumichi M, Sato Y, Kawashima M, Ishiguro-Watanabe M (2023). KEGG for taxonomy-based analysis of pathways and genomes. Nucleic Acids Res.

